# Supplementary material for: Adaptation and validation of the Weight Efficacy Lifestyle Questionnaire (WEL) in a Chilean sample
Source: PLoS One. 2024 Jan 31;19(1):e0293658. doi: 10.1371/journal.pone.0293658 (PMC10829987; doi:10.1371/journal.pone.0293658)
Supplement: S1 Table — (DOCX) [file pone.0293658.s001.docx]

**S1 Table.** **Weight Efficacy Lifestyle Questionnaire: English formulation**

| Physical and emotional discomfort | |
| --- | --- |
| 7. | I can resist eating when I am angry (or irritable) |
| 3. | I can resist eating when I feel physically run down |
| 6. | I can resist eating even when I have a headache |
| 12. | I can resist eating when I feel uncomfortable |
| 10. | I can resist eating when I have experienced failure |
| 4. | I can resist eating when I am depressed (or down) |
| External pression | |
| 2 | I can resist eating even when I have to say “no” to others |
| 1 | I can resist eating when I am anxious (nervous) |
| 11 | I can resist eating even when I think others will be upset if I don’t eat |
| 5 | I can resist eating even when I feel it’s impolite to refuse a second helping |
| 8 | I can resist eating even when others are pressuring me to eat |
