# Supplementary material for: Adaptation and validation of the Weight Efficacy Lifestyle Questionnaire (WEL) in a Chilean sample
Source: PLoS One. 2024 Jan 31;19(1):e0293658. doi: 10.1371/journal.pone.0293658 (PMC10829987; doi:10.1371/journal.pone.0293658)
Supplement: S2 Table — (DOCX) [file pone.0293658.s002.docx]

**S2** **Table. Weight Efficacy Lifestyle Questionnaire: Spanish formulation**

| Malestar físico y emocional | |
| --- | --- |
| 7. | Soy capaz de resistirme a comer cuando estoy enfadado/a (o irritable) |
| 3 | Soy capaz de resistirme a comer cuando me siento físicamente mal |
| 6 | Soy capaz de resistirme a comer incluso aunque tenga dolor de cabeza |
| 12 | Soy capaz de resistirme a comer cuando me siento mal |
| 10 | Soy capaz de resistirme a comer cuando he tenido un fracaso |
| 4 | Soy capaz de resistirme a comer cuando estoy deprimido/a (o desanimado/a) |
| Presión externa | |
| 2 | Soy capaz de resistirme a comer incluso aunque tenga que decirle que “no” a otros |
| 1 | Soy capaz de resistirme a comer cuando estoy ansioso/a (o nervioso/a) |
| 11 | Soy capaz de resistirme a comer incluso aunque piense que otros se van a molestar si no como |
| 5 | Soy capaz de resistirme a comer incluso aunque crea que es grosero rechazar un segundo ofrecimiento. |
| 8 | Soy capaz de resistirme a comer incluso aunque otros me presionen para que coma |
