# Supplementary material for: Adaptation and validation of the Weight Efficacy Lifestyle Questionnaire (WEL) in a Chilean sample
Source: PLoS One. 2024 Jan 31;19(1):e0293658. doi: 10.1371/journal.pone.0293658 (PMC10829987; doi:10.1371/journal.pone.0293658)
Supplement: Attachment — Submitted filename: Response to Reviewers_v2.docx [file pone.0293658.s003.docx]

2023, 31 July

Dear Editor

PLOS ONE

We would like to thank the reviewers for their in-depth review. Below we provide a detailed response to all your comments and suggestions. We hope to have addressed each of your concerns.

Please let us know any additional information that may be required.

Thank you very much for your support and guidance.

The authors

Answers to Reviewers Comments


Reviewer #1: Thanks for submitting this manuscript for review. Overall, I found it an interesting read, with potentially useful applications for public health research experiences and their subsequent actions. I have, however, a series of comments and suggestions that I will list below, following the order proposed by the authors:

First of all, the paper needs an extensive English writing edition.   Please refer to a certified/qualified translator or editor to proofread it.

Authors’ response: Thank you for your suggestion. The final version of the article was further revised by a certified translator.

The authors state that the objective of their paper is to "analyze   the psychometric properties of the Weight Efficacy Lifestyle   Questionnaire (WEL) in a sampling of Chilean adults from the general  population". Apart from being difficult to understand, the statement  does not refer to a validation process. In other words, the authors must be aware that analyzing the psychometric properties of a certain scale in a certain population does not imply validating it. Therefore, a conceptual and literature-based rationale is needed in order to clarify such a categorical error.

Authors’ response: Thank you for noticing this issue, we agreed with it, so the objective was modified in the Abstract and the main text as follows: *this study proposed to validate the WEL in a sample of Chilean adults from the general population*

- Apart from the aforementioned, the sample is too short for this purpose, and imbalanced in terms of sex/gender, age, and coverage.  Although it does not invalidate the study, the methodological issues  surrounding this set of facts must be better discussed and formulated
 in a revised manuscript.

Authors’ response: We totally agree with the comment and it is a limitation of the study. The implications of this limitation are discussed in more detail in lines 519 to 526: *On the other hand, the sample presents certain biases, for example, the greater participation of women, which may interfere with interpreting the results.* And: *Another aspect to consider is related to the differences in the level of self-efficacy according to sex. In a recent study in a gender-balanced sample using the WEL instrument, women showed lower levels of eating self-efficacy). The above, therefore, reinforces the fact that since this study has an unequal sample between men and women, the results should be analyzed with caution.*

- I feel the criteria used to choose and justify the criterion variables are concerning. While BMI is a good variable for this purpose, it varies over time; secondly, such prior attempts to lose weight are subject to different biases that are basically not controlled nor discussed throughout the paper. Please provide a  suitable justification for these technical shortcomings of the study.

Authors’ response: As mentioned, BMI and previous weight loss are discussed in more detail in lines 501 to 511 and 527 to 535*: Another limitation concerns BMI and previous attempts to lose weight as criterion variables. Regarding BMI, it is known that this is not the best method to evaluate the nutritional status of individuals, although it is recognized by the WHO as an international standard, it underestimates the total fat mass in people with lower body mass and may overestimate it in people with higher body mass (32). On the other hand, attempts to lose weight are influenced by several factors that were not considered in this article, so a larger sample with a longitudinal follow-up is needed to demonstrate this relationship.*

- Also, please make the data analysis plan more detailed and sequential in the methods section of the manuscript.

Author’s response: As suggested, the data analysis plan was detailed in the Method section.

- Line 223: This set of indices (e.g., an RMSEA under .080) does not   allow you to determine an "excellent" fit. Instead of using the rules  of thumb model, that is rather paracientific, I suggest the authors  referring to the March et al. (2004)-based indexes suggested in Table  2 of this paper: <https://pubmed.ncbi.nlm.nih.gov/34393528/>

Author’s response*:* A new analysis was performed that allowed a better fit, closer to what was suggested, with the following results: (X2(43)=77.33, p<0.001; X2/df=1.798; CFI=0.956; TLI=0.943; RMSEA=0.060; SMRM=0.054). Please see lines 318 to 320.

- Reliability analysis: Cronbach's alphas are not a strong/robust  indicator of it. At best, an indicator of internal consistency. Please calculate and report the CRIs for each factor.

Author’s Response: Composite Reliability was calculated for each factor and added to the manuscript. Please see lines 334 to 336.

- The complexity and robustness of the analyses and the methods used  are sometimes marginal. Why was this small-to-medium-size sample not  bootstrapped?

Author’s Response: The confirmatory factor analysis was robust to account for the reliability of the data. No analysis such as anova or t-analysis, was performed with small groups, so the observed differences are assumed reliable by the central limit theorem. Bootstrapped should be evaluated on a case-by-case basis for use in small samples, for example in the case of mediations, and in the case of median calculation, it has been indicated that alternative methods are better to be used. Please see: Zhang M, Liu X, Wang Y, Wang X. Parameter distribution characteristics of material fatigue life using improved bootstrap method. International Journal of Damage Mechanics. 2019;28(5):772-93.

<https://journals.sagepub.com/doi/10.1177/1056789518792658>

- Most of the study limitations (apart from the aforementioned) are clearly under-acknowledged and under-discussed with methodological  sources as support.

Author’s Response: Thank you for raising this point. As suggested we have strengthened the Limitations of the study. Please see pages 21 to 22.

- Please kindly translate the figure legends and captions, uniformly.

Author’s Response: we have checked and corrected figure legends and captions according to Instructions for Authors.

- Again, please make sure to avoid confusing a psychometrical  assesment with a validation. Conclusions are formulated in terms of  the first, but it is always confusing when referring to the title of  the paper, for which authors need some extra steps.

Author’s Response: as mentioned above, we agreed with the comment and modified the objective. This new version was in accordance with the title.

- Although in view of the few analyses used the first conclusion seems  adequate (except for the term "validation", the fact that the authors  invite to use it in clinical contexts is totally out of scope (line  448). What are the background or prospects in these regards? Your  study does not provide them. Please elaborate and reformulate.

Author’s Response: the suggestion is accepted. Conclusions were modified considering that this study addressed the general population; thus, self-efficacy can be measured in this group but not in people in treatment to lose weight. Please see lines 576 to 582.

- A suitable database is needed for statistical checks among referees.
  Could you provide it?

Author’s Response: In the Data Availability statement, we have added the link where the database is hosted: https://osf.io/s9uzf/?view_only=3380828e78e44cc790c12714faeb00a4

**Reviewer #2:**

The aim of the study was to analyze the psychometric properties of the Weight Efficacy Lifestyle Questionnaire (WEL) in a sample of 469 Chilean adults (69.08% were female, mean age=38,02; SD=10,31). The study is interesting; psychometric analyses were used; the article is well written. Some parts of the manuscript could be clarified. I have
made a report and I hope it will be useful to help the authors in the review.

Abstract
- “The sample included 469 people from the general population in Chile…” Revise to: “A total of 469 individuals (69.08% were female, mean age=38,02; SD=10,31) participated in study”.

Author’s Response: the suggestion is accepted and the modifications were made.

Background
- The background used to justify the study is robust and in line with the aim.

Material and Methods
- I would like to know how the authors calculated the sample size needed to perform the analyses. Adding this to the first Methods section is important.

Author’s Response: The suggestion is accepted and the modifications were made. You can find this information on lines 162 to 166.

- “An instrumental design was used that was geared to the development of psychometric tests and instruments, including their adaptation”.
Please provide the exact reference of the instrument designer that was used.

Author’s Response: We agree that was not clear enough the study design, which in this case refers to an instrumental design. To clarify the above, the reference in which the above is specified is added on lines155-159-.

Results

- “The first factor was designated as "Physical and emotional distress…The second factor was termed "External pressure" and includes 5 items…”. Please advise that these labels were created by the authors.

Author’s Response: the suggestion is accepted and the modifications were added. You can find this information on lines 304 to 309.

- “A CFA was performed to test the one- and two-factor structure…”. Why did the authors test the one-factor structure?

Author’s Response: We test the one-factor structure because in the analysis with Velicer's MAP we obtained a minimum of 0.03 with 1 factor, the same as in Horn's Parallel Analysis. The aforementioned was indicated in lines 289 to294.

- “Due to the above, it was decided that a new confirmatory analysis would be performed without including Item b4 in Factor 1”. Why was item b4 excluded from the test? Because of the low factor loading? Why was it not also tested without item b9?

Author’s Response: Thank you for your suggestion. New analyses are performed, one excluding item b4 and another one excluding item b9. The best fit is obtained without item b9, for this reason, it is decided to perform all the analyses again without that item. The details of the aforementioned can be found between lines 322 and 342.

Discussion

- “Regarding the reliability analysis, the instrument shows high internal consistency, with similar results to those reported in other validations (16,17)”. What does that mean? It would be important to explore this result further.

Author’s Response: Details of your suggestion are provided between lines 480 and 484

- There are very short paragraphs in this section. For example, the fourth paragraph consists of only one sentence. I suggest reviewing the Discussion to avoid this. Another suggestion is to create a subsection (e.g., Limitations and Strengths) to bring together the last few
paragraphs.

Author’s Response: the suggestion was accepted and the modifications were added. Please see Limitations which were discussed in more detail in lines 519 to 526:

Conclusion

- The conclusion is unclear and "inappropriate". It was not the evaluation of the Chilean version of the WEL that showed adequate psychometric properties; in fact, the study found that there was validity and reliability of the Chilean data collected from the WEL.

Author’s Response:

Furthermore, how do the study results clearly support the use of WEL in clinical context if the sample was non-clinical? The fact that there are people with obesity in the sample does not mean that they are" clinical people". I suggest reviewing that section.

Author’s Response the suggestion is accepted and the modifications were made in the Conclusion emphasizing that the sample was a general population thus, no conclusions can be made about any other particular groups.
