# Supplementary material for: Adaptation and validation of the Weight Efficacy Lifestyle Questionnaire (WEL) in a Chilean sample
Source: PLoS One. 2024 Jan 31;19(1):e0293658. doi: 10.1371/journal.pone.0293658 (PMC10829987; doi:10.1371/journal.pone.0293658)
Supplement: Attachment — Submitted filename: Response to Reviewers.docx [file pone.0293658.s004.docx]

October 3, 2023

Answers to Reviewers' Comments


*Reviewer #1: Thanks for your revisions and amendments. I have some further comments, which should be addressed by the authors for their resubmission:

I have been cross-checking the survey data, finding that there are some potential bias sources that, although not under authors' control, must be addressed in the manuscript.*

*- Please add a suitable elaboration on how missing data were handled, and what kind of data were mainly omitted. Also, please discuss this > in the light of both the current study settings and any relevant technical source contained in literature.*

Authors answer: No data imputation technique was necessary because the R program only performed the analyses with complete data. This information is found on lines 225 and 226 of the manuscript with track changes.

*- Also, did you assess invariance? I guess there are probably sex and other categorical factor-based invariance issues. In case you tested > it, please add it to the paper (briefly). If not, you can add this as a technical limitation of the study.*

Although it would have been good to calculate invariance, the sample is unbalanced between men and women, and the presence of other covariates has not been controlled. This will be reported as a limitation of the study and you can find it on lines 476 to 479 of the manuscript with track changes.

*- Thanks for adding the composite reliability analysis outcomes. It improves a lot the assumptions related to reliability of your paper. Please also make this evident (and literature-supported) in the discussion of the manuscript.*

The reviewer's suggestion was accepted and the implications of the composite reliability results were included in the Discussion. This information is found on lines 417 to 423 of the manuscript with track changes.

*- In the appendix, please add another column with the most suitable translation of the items to the English (given that most of your readers are supposed to be non-Spanish but English speakers).*

We appreciate the reviewer's suggestion and have added the English translation of the scale. This information is found on lines 628 to 629 of the manuscript with track changes.

*- In conclusions section, the sentence "More studies are needed to (...)" is not a conclusion of your study (you did not address it from an empirical perspective). Therefore, please raise it as a practical implication, in a subsequent paragraph.*

We appreciate the reviewer's suggestion and incorporate it into the manuscript. This information is found on line 493 of the manuscript with track changes.

- Finally, and as a matter of courtesy with your reviewers, please improve the way of track changes in a revised manuscript version. Using strikethrough text makes it very difficult to follow along. Better use the highlight tool, and/or a different color font.

We apologize for the confusing nature of the above manuscript. Changes are highlighted in yellow and deleted in red.

Reviewer #2: The authors reported that they agreed with all my comments and revised the manuscript; however, I have not been able to find the changes in the manuscript. The number of lines mentioned by the authors in the response letter does not correspond to the numbers in the manuscript. In order for me to properly proofread the manuscript, authors need to highlight changes to the text (eg, using yellow color).

We apologize for the confusing nature of the above manuscript. Changes are highlighted in yellow and deleted in red.
